# Rapeseed protein-derived peptides, LY, RALP, and GHS, modulates key enzymes and intermediate products of renin–angiotensin system pathway in spontaneously hypertensive rat

**DOI:** 10.1038/s41538-018-0033-5

**Published:** 2019-01-17

**Authors:** Rong He, Yi-Jie Yang, Zhigao Wang, Chang-rui Xing, Jian Yuan, Li-Feng Wang, Chibuike Udenigwe, Xing-Rong Ju

**Affiliations:** 1 College of Food Science and Engineering/Collaborative Innovation Center for Modern Grain Circulation and Safety/Key Laboratory of Grains and Oils Quality Control and Processing, Nanjing University of Finance and Economics, Nanjing, 210023 China; 2 School of Food Science and Technology, Jiangnan University, Wuxi, 214122 China; 3 School of Nutrition Sciences, University of Ottawa, Ottawa, ON K1H 8L1 Canada

**Keywords:** Proteins, Molecular biology

## Abstract

Rapeseed proteins are a rich source of bioactive peptides. LY, RALP and GHS were previously identified from rapeseed protein hydrolysates as potent ACE and renin inhibiting peptides. In this study, the rapeseed peptides were individually evaluated for their molecular mechanisms and regulatory effects on components of the renin–angiotensin system in spontaneously hypertensive rats (SHR), including the mRNA and/or protein levels of angiotensin-converting enzyme (ACE), renin, ACE2, angiotensin II and angiotensin-(1–7) in myocardial tissues. Oral administration of 30 mg peptides/kg body weight every 2 days for five weeks significantly decreased the systolic blood pressure and the myocardial mRNA and protein levels of ACE and renin in SHR. LY, RALP and GHS also increased the expression of ACE2, angiotensin-(1-7) and Mas receptor levels, which may have mediated their antihypertensive activity. Dipeptide LY also inhibited angiotensin II protein expression in the heart tissue. Taken together, the finding demonstrates the multi-target physiological effects of the rapeseed peptides, beyond ACE and renin inhibition, which enhances knowledge of the antihypertensive mechanisms of food protein-derived peptides.

## Bioactives: Food derived peptides act as antihypertensives

There is growing interest in developing food originating antihypertensive peptides. The rapeseed protein hydrolysates LY, RALP and GHS are known as inhibitory peptides, capable of lowering the blood pressure. Now Xing-Rong Ju from Nanjing University of Finance and Economics and coworkers evaluated their regulatory effects from the molecular level. And they found the inhibitory roles of LY, RALP and GHS were multi-targeted at gene and/or protein levels. The decrease of the systolic blood pressure after their five weeks’ oral administration might be attributed to the increased expression of ACE2. Among these three peptides, LY was found to exhibit the most effect which may be due to its stronger inhibition on the expression of Ang II protein in the heart tissue. These remain to be further explored.

## Introduction

Hypertension is a major risk factor for developing cardiovascular disease, which is estimated to result in 23.3 million deaths in 2030.^1^ The renin-angiotensin system (RAS) is an important pathway for the regulation of blood pressure and other cardiovascular processes.^2^ RAS is also associated with hypertension in spontaneously hypertensive rats (SHR),^3^ which is widely used in studying the antihypertensive effects and mechanisms of exogenous compounds. In the RAS pathway, renin, the first and rate-limiting enzyme, converts angiotensinogen to angiotensin I (Ang I), which is inactive. Ang I can then be hydrolyzed by angiotensin I-converting enzyme (ACE) to release a potent vasopressor octapeptide, angiotensin II (Ang II).^4^ Moreover, angiotensin-converting enzyme 2 (ACE2), a homolog of ACE, was subsequently discovered to catalyze the conversion of Ang II into angiotensin-(1–7) (Ang-(1–7)).^5^ ACE2 also hydrolyzes Ang I to form Ang (1–9), which can then be converted to Ang (1–7) by ACE. Ang-(1–7) binds to G-protein-coupled receptor, Mas, to induce vasodilation, which antagonizes the vascular effects of Ang II.^6^ Excessive activity of RAS will contribute to abnormally high Ang II level and reduction rates of vasodilation, which is one of the major contributors to hypertension.

Traditional drug treatments have involved the use of compounds that inhibit the activities of ACE or renin as well as those that reduce binding of Ang II to its receptors or those that increase NO level in the vascular endothelium.^7,8^ However, use of these drugs may lead to negative side effects such as dry cough, edema, skin rashes, and erectile dysfunction.^9–11^ Consequently, over the last two decades, there has been increased interest in the development of antihypertensive peptides from food proteins, primarily because of the low cost and low toxicity associated with the products. However, low bioavailability of peptides in vivo is one of the major limitations to their development as antihypertensive agents. Emerging studies are focusing on enhancing peptide bioavailability and elucidating if the blood pressure-lowering effect of peptides is mediated through the expression of major RAS genes and proteins.^12^ For instance, egg ovotransferrin-derived ACE inhibitory peptide IRW increased the mRNA expression of ACE2 in SHR.^13^ Renal mRNA expression of ACE and renin were also reduced when pea protein hydrolysate was fed to SHR.^14^ Similar results were found for egg protein-derived peptides, QIGLF and RVPSL.^15,16^ Furthermore, lactoferrin-derived peptide RPYL was reported to inhibit Ang II activity by blocking its binding to angiotensin receptor 1 in rabbit carotid artery.^17^ Therefore, the RAS regulatory mechanisms of peptides is more extensive than the widely studied inhibition of ACE activity, and many of the peptides may be exerting their effects on the RAS proteins at the transcriptional and post-transcriptional levels.

In our previous study, dual ACE and renin inhibitory peptides, LY, RALP, and GHS, were identified from rapeseed protein hydrolysate that lowered blood pressure when orally administrated to SHR over a short period.^18–20^ Moreover, LY resisted hydrolysis when transported across Caco-2 cell monolayers, whereas RALP was hydrolyzed into smaller fragments, which also inhibited ACE and renin activities in vitro.^21^ Based on the previous findings, the objectives of this study were to evaluate the long-term antihypertensive effects of rapeseed peptides, LY, RALP, and GHS, in SHR and their molecular mechanisms in regulating major RAS genes and proteins in the rat myocardial tissue.

## Results

### Long-term antihypertensive activities of LY, RALP, and GHS in SHR

**Fig. 1:**
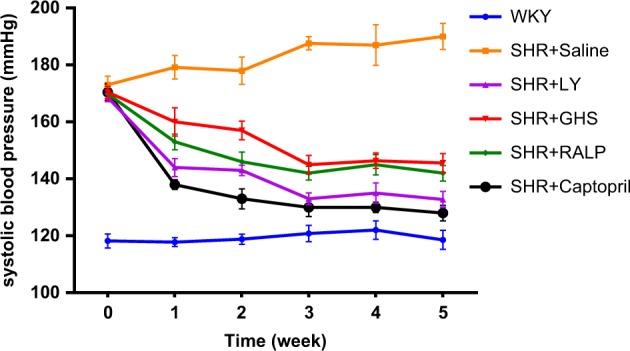
Long-term antihypertensive effects of peptides LY, RALP, GHS, and captopril in spontaneously hypertensive rats (SHRs): Change in the systolic blood pressure (SBP) of SHRs post-administration with saline, peptides and captopril, respectively. Bars (mean ± SD, *n* = 5) with different alphabets have mean values that are significant different (*p* < 0.05)

In order to investigate the long-term antihypertensive effects of LY, RALP, and GHS in SHRs, the weekly blood pressures of rats in the different groups were compared after peptide administration from week 1 to week 5. As shown in Fig. 1, systolic blood pressure (SBP) of the negative (SHR) group that received saline increased from 173 to 190 mm Hg after 5 weeks. Peptides, LY, RALP, and GHS, showed antihypertensive property in vivo based on their effect in lowering SBP of SHR from about 173 mm Hg to 132, 142, and 145 mm Hg, respectively after 5 weeks. The blood pressure-lowering effects of the peptides were lower than that of an antihypertensive drug, captopril, which lowered SBP to 128 mm Hg under the same condition. There was no significant change in SBP of the WKY rats that received saline during the study.

### ACE, renin, and ACE2 mRNA expression levels in the rat myocardial tissue

**Fig. 2:**
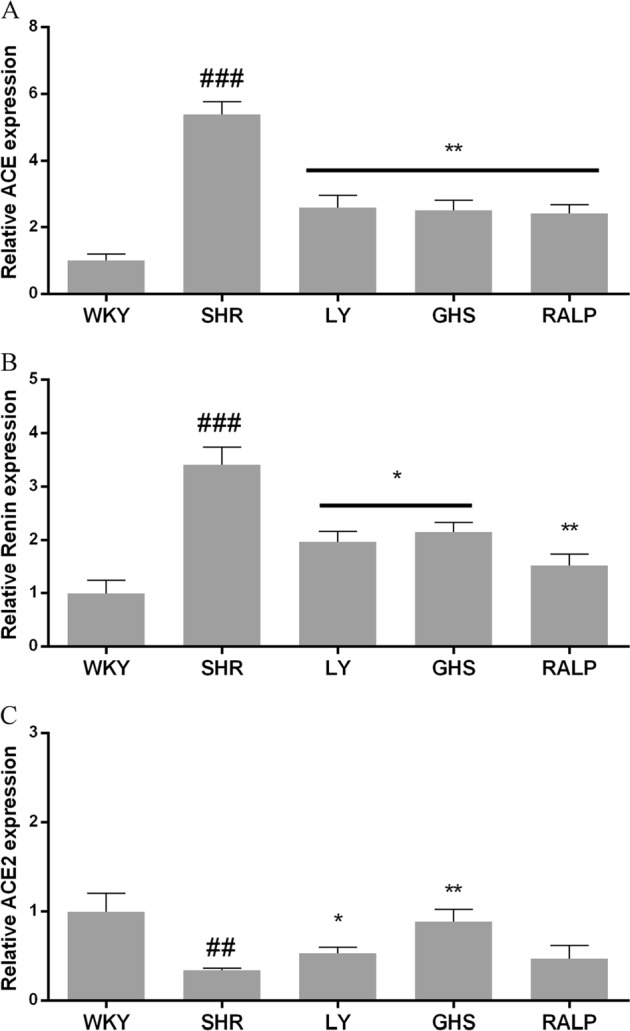
Effects of three peptides LY, RALP, and GHS on the mRNA expression of **a** ACE, **b** renin, and **c** ACE2 in myocardium tissue of spontaneously hypertensive rats (SHRs) compared with that of WKY rats. Data are expressed as mean ± SD of five independent experiments, normalized to their loading control. ## and ### indicates *p* < 0.01 and *p* < 0.001, respectively, as compared to the WKY group. * and ** indicates *p* < 0.05 and *p* *<* 0.01, respectively, as compared to the SHR group

ACE, renin, and ACE2 are major enzymes of RAS. Therefore, the level of expression of their mRNA can be used to indirectly determine the effects of exogenous compounds on RAS. As shown in Fig. 2a, the administration of LY, RALP, and GHS to SHR for 5 weeks led to a significantly lower level of ACE mRNA expression in the myocardial tissue. Similar results were found for renin mRNA expression in the rat tissue (Fig. 2b). Furthermore, as shown in Fig. 2c, administration of LY and GHS resulted in a higher level (*p* < 0.05) of ACE2 mRNA in SHRs, whereas RALP had no significant effect (*p* > 0.05). Interestingly, GHS had the same ACE2 mRNA level as the normotensive WKY group.

### Regulation of ACE, renin, and ACE2 protein expression by LY, RALP, and GHS

**Fig. 3:**
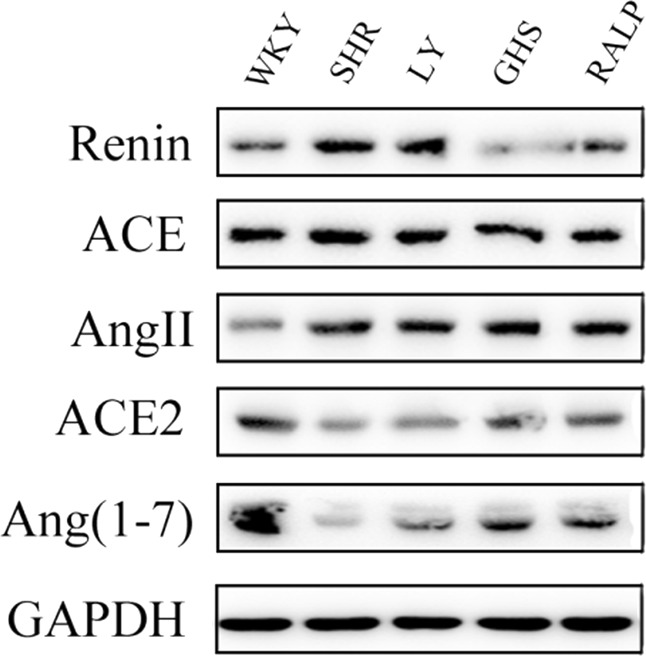
Western blot analysis of the three peptides’ effects on renin, ACE, Ang II, ACE2, and Ang (1–7) in myocardium tissue of spontaneously hypertensive rats (SHRs) compared with that of WKY rats. GAPDH was used as internal control

**Fig. 4:**
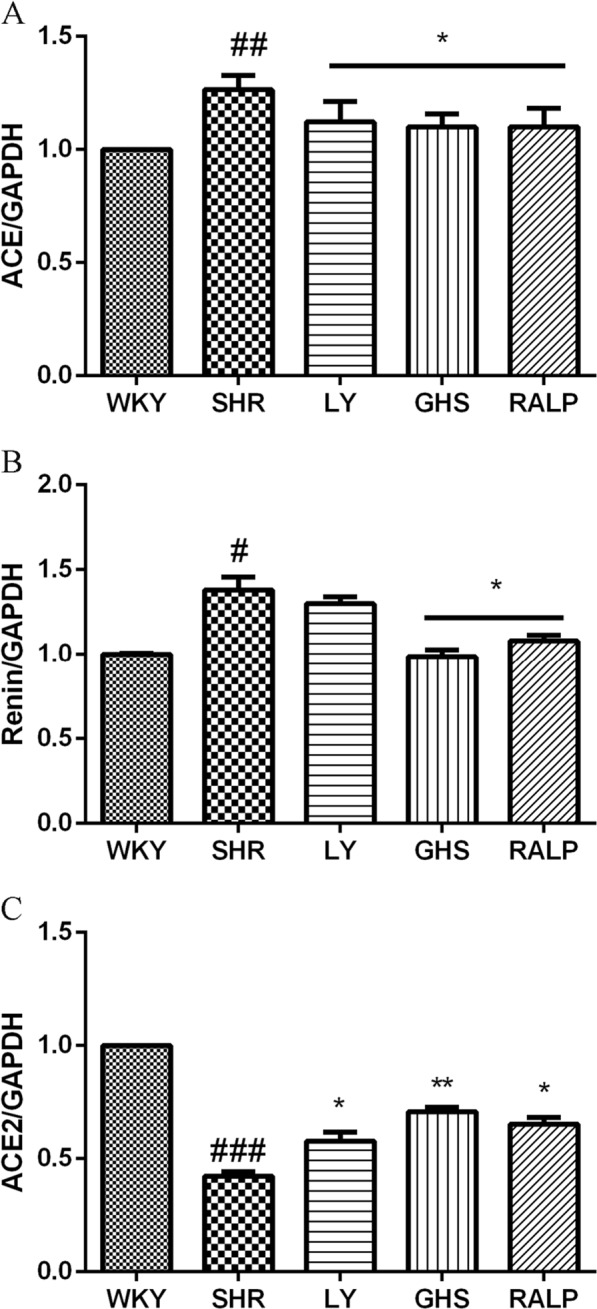
Effects of three peptides LY, RALP, and GHS on protein expression of **a** ACE, **b** renin, and **c** ACE2 in myocardium tissue of spontaneously hypertensive rats (SHRs) compared with that of WKY rats. Data are expressed as mean ± SD of five independent experiments, normalized to their loading control. #, ##, and ### indicates *p* < 0.05, *p* < 0.01, and *p* < 0.001, respectively, as compared to the WKY group. * and ** indicates *p* < 0.05 and *p* *<* 0.01, respectively, as compared to the SHR group

In order to evaluate whether the decrease of ACE, renin, and ACE2 mRNA expression was associated with protein changes, western blot analysis was performed. As shown in Fig. 3, ACE, renin, and ACE2 protein levels differed significantly between the control and peptide groups, which is in agreement with the mRNA data. As shown in Fig. 4a, the three peptides inhibited ACE protein expression significantly when compared to the SHR control group (*p* < 0.05). Renin protein level was also lower in SHR that received GHS and RALP groups, down to the level observed for WKY rats, but not in the LY-treated SHR group (Fig. 4b). Lastly, all the peptides resulted in a higher level of ACE2 protein in the hypertensive rats compared to the SHR control group, although the normotensive WKY rats had significantly higher ACE2 level (Fig. 4c).

### Effect of LY, RALP, and GHS on Ang II, Mas, and Ang-(1–7)

**Fig. 5:**
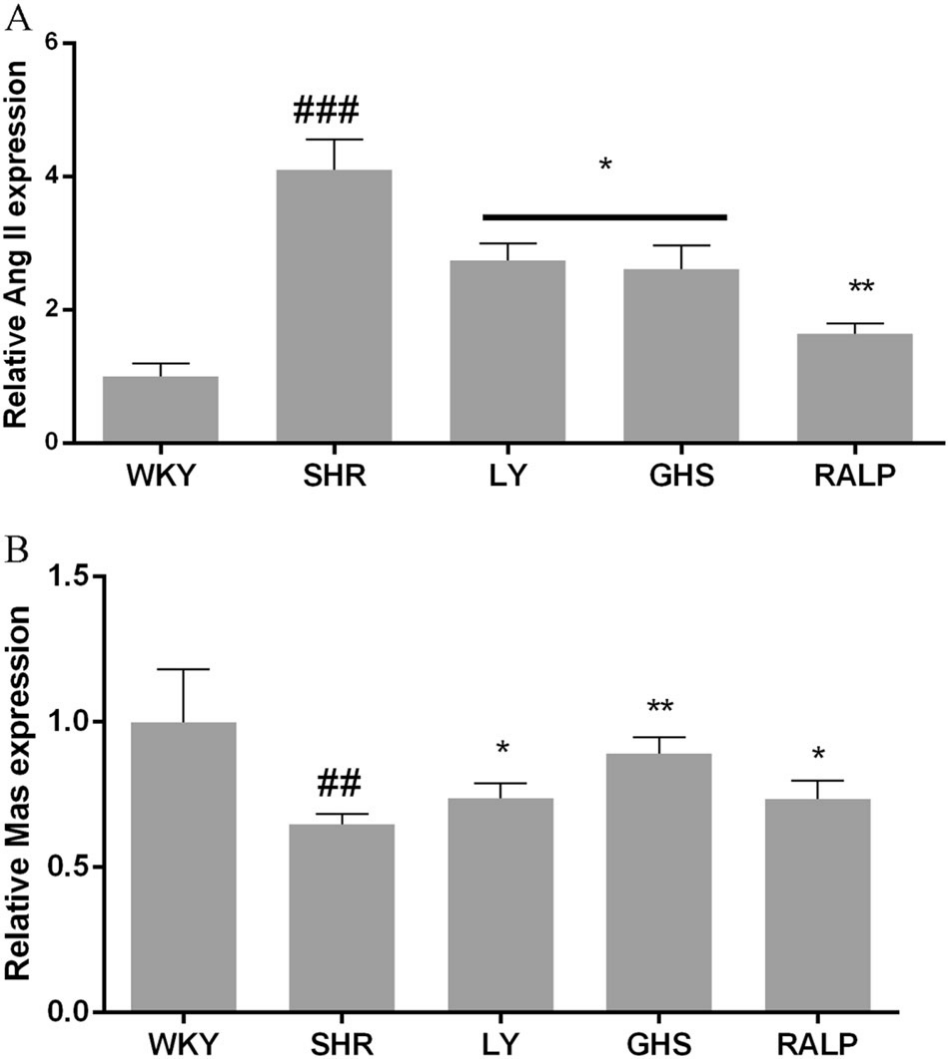
Effects of three peptides LY, RALP, and GHS on the mRNA expression of **a** Ang II and **b** Mas receptor in myocardium tissue of spontaneously hypertensive rats (SHRs) compared with that of WKY rats. Data are expressed as mean ± SD of five independent experiments, normalized to their loading control. ## and ### indicates *p* < 0.01 and *p* < 0.001, respectively, as compared to the WKY group. * and ** indicates *p* < 0.05 and *p* *<* 0.01, respectively, as compared to the SHR group

**Fig. 6:**
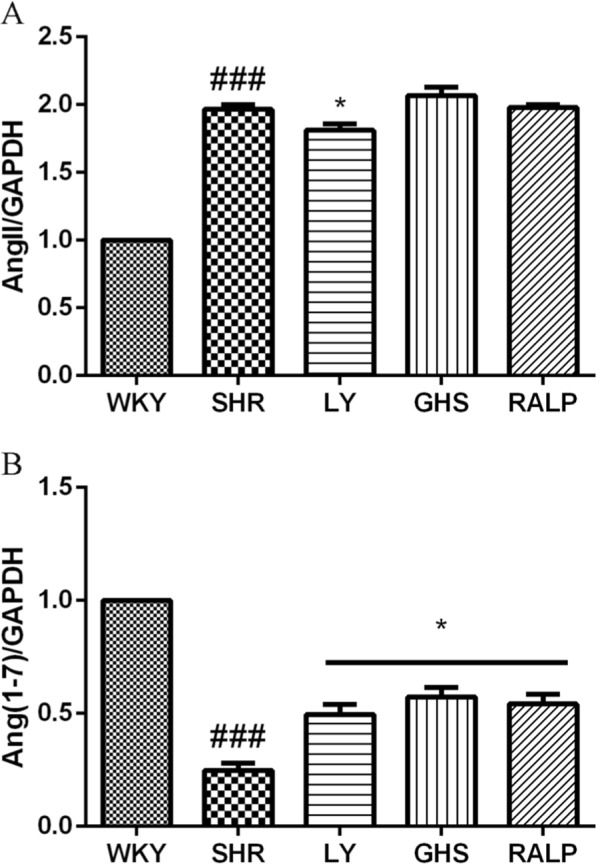
Effects of three peptides LY, RALP, and GHS on protein expression of **a** Ang II and **b** Ang (1–7) in myocardium tissue of spontaneously hypertensive rats (SHRs) compared with that of WKY rats. Data are expressed as mean ± SD of five independent experiments, normalized to their loading control. ### indicates *p* < 0.001 as compared to the WKY group. * indicates *p* < 0.05 as compared to the SHR group

The regulatory effects of the rapeseed peptides on key RAS products, Ang II and Ang-(1–7), at the gene and/or protein levels were evaluated in the rat myocardium. As shown in Fig. 5a, mRNA level of Ang II was lower in the SHR group that received the peptides when compared to the SHR group. Moreover, mRNA level of Mas receptor was higher in the peptide-treated SHR groups compared to the SHR control group (Fig. 5b). Similarly, as shown in Fig. 6b, Ang-(1–7) protein level was higher in the rat myocardium at 5-week peptide treatment, which is consistent with the pattern observed for its Mas receptor gene level. Interestingly, only LY downregulated Ang II protein level significantly (*p* < 0.05), of all the peptide treatments, relative to the SHR control group (Fig. 6a). This pattern differs from the result observed for Ang II mRNA level with the peptide treatments.

## Discussion

Bioactive peptides derived from food are promising candidates for the prevention or treatment of chronic diseases, such as hypertension, obesity, cancer, and diabetes, because of their diverse structural features.^22,23^ Rapeseed meal, which is a byproduct of oil extraction, contains up to 50% protein on a dry weight basis and has a diverse amino acid composition for generating bioactive peptides.^24^ Most food protein-derived antihypertensive peptides were initially thought to exhibit their effects through ACE inhibition.^25^ Our previous study showed that rapeseed protein-derived peptides LY, RALP, and GHS, which can cross Caco-2 intestinal monolayer intact, inhibit the enzymatic activity of ACE and also other RAS components such as renin.^21^ However, there is a dearth of information on the biological mechanisms of the peptides in vivo, especially after a long-term administration.

In the present study, oral administration of saline did not lower the SBP of SHR. The substantial SBP-lowering effect observed for peptides, LY, RALP, and GHS, after 5 weeks suggests that they can potentially be used as antihypertensive nutraceuticals. On the basis of the 30 mg peptide/kg body weight administrated to the rats, a 70 kg human would need to consume ~ 2 g of the peptides every 2 days to maintain sustained SBP decrease.^20^ This effective SBP reducing dose is higher than the effective dose of captopril, but it can be achieved through twice-daily consumption of one 500 mg tablet of the peptides. Moreover, the SBP reducing activities of LY, RALP, and GHS were higher than that of egg-derived QILGF, whose SBP-lowering activity was evaluated at a higher daily dose of 50 mg/kg after 4 weeks.^15^ Similarly, the rapeseed peptides showed superior long-term antihypertensive effect in SHR when compared with egg-derived RVPSL.^16^ Recently a long-term study was carried out where the tripeptide GVR was orally administered to SHRs with 50 and 100 mg/kg bw for 21 days and the SBP reduction was observed significantly by 23 and 30 mm Hg, respectively.^26^ Although the SBP-lowering effects of the rapeseed peptides were lower than that of captopril, their putative safety, as naturally derived compounds, makes them attractive for further evaluation as physiologically active agents for preventing hypertension.

Renin is the first enzyme in RAS and it converts angiotensinogen to inactive decapeptide, Ang I. Our results showed that renin expression in heart tissue of SHR was much higher than that of normotensive WKY. Oral administration of peptides LY, RALP, and GHS downregulated renin expression, at both the gene and protein levels, compared to the SHR control group. Moreover, renin mRNA and protein expression were inhibited significantly by the peptides, with RALP and GHS reducing myocardial renin level close to the protein level in the normotensive WKY rats. This result follows the same pattern as our previous observation in vitro that RALP had the highest renin inhibitory activity with IC_50_ of 0.97 mM.^19^ A few studies have demonstrated the effects of long-term intake of food peptides on renin in vivo. For instance, intake of a pea protein hydrolysate (0.5% and 1% of diet) for 8 weeks inhibited renal renin mRNA by about 50% in Han:SPRD-cy rats, a chronic kidney disease model with hypertension.^14^ Similarly, egg protein-derived peptide, RVPSL, also decreased kidney renin mRNA expression after 4 weeks of daily intake.^16^ On the contrary, whey protein-derived dipeptide, IW, increased renin mRNA expression in the kidney.^27^ This discrepancy could be due to the difference in peptide structure and SHR tissues, in addition to other unknown factors. Considering that renin controls the rate-limiting step of the RAS pathway, it is critical to consider the pronounced effects of RALP and GHS on renin enzymatic activity in vitro, and its myocardial mRNA and protein levels, as a major antihypertensive mechanism of the peptides.

ACE also plays a critical role in RAS as it is responsible for the conversion of Ang I to vasoconstrictor Ang II, the production of Ang (1–7) from Ang (1–9), and the inactivation of vasodilator, bradykinin. In the present study, oral administration of the rapeseed peptides LY, RALP, and GHS reduced ACE expression at both the mRNA and protein levels. Our previous study detected minor quantity of RALP, and large amounts of LY and RALP fragments (ALP, LP, and RA) after uptake to the basolateral side of Caco-2 cell monolayers; the RALP fragments still retained ACE and renin inhibitory activities.^21^ Moreover, it has been reported in our previous study^28^ that LY had strong binding effect on ACE molecular by intrinsic fluorescence intensity analysis and molecular docking study confirmed the rapeseed peptides inhibited renin and ACE activities mostly through binding to enzyme active site or non-active site and forming extensive H-bonds that distorted the normal configuration required for catalysis. Thus, the physiological effects on the myocardial gene and protein levels could be due to the intact rapeseed peptides or their active fragments. Others have also reported that food protein peptides and peptides could reduce ACE mRNA expression, including QILGF,^15^ RVPSL,^16^ blue mussel protein hydrolysate^29^, and pea protein hydrolysate.^14^ The additional effect of the rapeseed peptides on ACE protein level could be a direct consequence of their effect on ACE mRNA in the rat myocardium.

ACE2 is a homolog of ACE that cleaves the C-terminal phenylalanyl residue of Ang II to form Ang-(1–7). It also hydrolyzes Ang I into Ang-(1–9), which is then converted into Ang-(1–7) by ACE.^12^ Results from our study showed that oral administration of the rapeseed peptides to SHR for 5 weeks, the mRNA and protein expression of ACE2 were increased compared to the SHR control group, which demonstrates the multi-targeted inhibitory roles of the peptides in modulating RAS components and hypertension in the rats. In fact, the reduction of Ang II and the increased of Ang-(1–7) observed for the rapeseed peptides could have been mediated, at least partially, through the increased myocardial ACE2 expression. Interestingly, peptide GHS, which contains predominantly hydrophilic amino acid residues, exhibited the most pronounced effects on ACE2 gene and protein, even when it had the least SBP-lowering effect in SHR. Egg ovotransferrin-derived peptide IRW was the first ACE inhibitory peptide reported to increase ACE2 expression in mesenteric artery of SHR.^13^ A previous study suggested that milk-derived ACE-inhibiting peptide, IPP, might have exerted additional antihypertensive effects through activation of the ACE2-Ang_1-7_-Mas axis of RAS.^30^ However, whether all the ACE inhibitory peptides, or their fragments, could activate or upregulate ACE2 in vivo still need further investigation. Based on the findings, we have demonstrated that food protein-derived ACE and renin dual inhibitory peptides could exert antihypertensive effects by increasing both the gene and protein levels of ACE2.

Ang II is a potent vasoconstrictor that is directly responsible for triggering the physiological process that leads to hypertension. Ang II exerts its biological effects through the binding to two G-protein-coupled receptors, AT1 and AT2. An increased level of Ang II would contribute to an increase in blood pressure and potentially hypertension. In contrast, Ang-(1–7) plays an opposite role in the regulation of blood pressure.^31^ Specifically, Ang-(1–7) binds Mas, a G-protein coupled receptor, to activate a signaling pathway that leads to vasodilation, thereby counteracting the effect of Ang II. Our study demonstrated the elevated level of Ang-(1–7) protein after oral administration of the rapeseed peptides, compared to the SHR control group, which might have resulted from the upregulation of ACE2 gene and protein levels. Moreover, Mas receptor mRNA expression was also increased by the peptides. This result indicated that ACE2-Ang_1-7_-mas axis is another possible pathway through which the ACE/renin dual inhibitory rapeseed peptides could have mediated their antihypertensive effects. Interestingly, the ACE2-Ang_1-7_-Mas axis also significantly inhibits pancreatitis by inhibition of the p38 MAPK/NF-κB signaling pathway.^32^ However, although Ang II mRNA expression was inhibited significantly by the three peptides, only LY decreased Ang II protein level in the myocardium when compared to the SHR that received only saline. Increasing evidence suggests that Ang II could also be generated via multiple pathways that may not be blocked by classic ACE inhibitors, such as the chymase-dependent process.^33–35^ Consequently, it is possible that alternative Ang II synthetic processes in myocardial tissues of the hypertensive rats also influenced the total Ang II protein level in our study.

In conclusion, we have demonstrated in this study that oral administration of rapeseed-derived dual ACE/renin inhibitory peptides, LY, RALP, and GHS, to SHR for 5 weeks modulated the expression of myocardial renin, ACE, ACE2, Ang II, Ang-(1–7), and Mas receptor at gene and/or protein levels. The peptides also exhibited long-term effects in lowering of systolic blood pressure in SHR with LY showing the most potent effect. RALP and GHS had more pronounced effects on renin and only LY inhibited the expression of Ang II protein. All the three peptides increased ACE2 level, which is emerging as a new antihypertensive mechanism of ACE/renin inhibiting peptides. It’s likely that small molecular size antihypertensive peptides are easier to be absorbed intestinal. Moreover, it is possible that the peptide functioned via a concerted mechanism involving different other mechanisms, such as increase expression of endothelial nitric oxide synthase, release of nitric oxide and effects on vascular smooth muscle cells. Results from this study will enhance knowledge of the regulatory mechanisms of antihypertensive food-derived peptides. Future study should concentrate on their receptors and relate with structural sequences of peptides.

## Materials and methods

### Materials and reagents

TRIzol reagent and DNase I (RNase-free) were purchased from Invitrogen (Shanghai, China). PrimeScript RT reagent Kit was purchased from Takara Biomedical Technology Co., Ltd. (Beijing, China). SYBR green was purchased from Bio-Rad Laboratories (Shanghai, China). BCA protein assay kit was purchased from Beyotime Institute of Biotechnology (Shanghai, China). RIPA lysis buffer and pheylmethylsulfonyl fluoride (PMSF) were purchased from Solarbio Science and Technology Co., Ltd. (Beijing, China). Primary antibodies against ACE (sc-23908), ACE2 (sc-390851), renin (sc-137252,) and Ang II (sc-74402) as well as secondary antibodies and anti-GAPDH were purchased from Santa Cruz Biotechnology Inc. (Shanghai, China). Antibody Ang (1–7) (139239) was purchased from USBiological Life Science. Peptides, LY, RALP and GHS, were synthesized to > 95% purity by Synthgene Biological Technology Co., Ltd (Nanjing, China).

### Determination of antihypertensive activity

A total of 25 adult (10-weeks-old) male SHR and five adult Wister-Kyoto rats (WKY) of the same age were obtained from Beijing Vital River Laboratory Animal Technology Co., Ltd. (Beijing China). Animal care and euthanasia were carried out with the approval of the Institutional Animal Care and Use Committee (IACUC). The rats were acclimatized for a week in a pathogen-free animal housing facility at Nanjing Synthgene Biological Technology Company under a 12-h day and night cycle at 21 ℃. The rats were fed a regular chow diet and tap water, and were randomly divided into six groups with five rats per group: (i) 0.9% saline for WKY, (ii) 0.9% saline for SHR, (iii) LY for SHR, (iv) RALP for SHR, (v) GHS for SHR, and (vi) captopril for SHR. The peptides (30 mg/kg body weight), and captopril (10 mg/kg body weight) were dissolved in phosphate buffered saline and orally administrated to rats every other day as previously described.^19^ The rats from different groups were orally administrated 1 mL of corresponding solution every other day and the SBP was measured for 5 weeks (same time each week) using the tail-cuff method.

### RNA extraction and quantification

Total RNA in cardiac muscle tissue was isolated using Invitrogen TRIzol following to the manufacturer’s procedure. Briefly, cardiac muscle tissue of the rats was collected, mixed with 1 mL TRIzol reagent, and homogenized for 10 min. Then, 0.2 mL chloroform was added and the mixture was centrifuged at 14,000 ⨯ *g* for 15 min at 4 ℃. The upper aqueous phase was collected, 0.5 mL isopropanol was added to it, and the mixture was kept for 10 min followed by centrifugation for 10 min. The supernatant was discarded and the precipitate was washed twice with 75% ethanol before centrifugation. The washed RNA was air dried prior to further analysis. DNase I was then used to eliminate the interference of genomic DNA. The cDNA was generated using PrimeScript RT reagent kit and PCR reaction was then performed in a 2720 Thermal Cycler (Applied Biosystems; Canada). Primer sequences used in this study are shown in Table 1. Quantitative real-time PCR was performed using the Roto-gene 6000 real-time PCR machine (Corbett; Australia). The amplification cycles were carried out at 95 ℃ for 2 min and 60 ℃ for 15 s. After 40 cycles, the PCR products were separated by electrophoresis on a 1.5% agarose gel for 30 min at 100 V. For analysis, cycle threshold (Ct) values were calculated for each sample. Relative quantification was achieved by the comparative 2^−ΔΔCt^ method.^36^

**Table 1:** Nucleotide sequences of PCR primers

Gene name	Orientation	Primers sequences (5ʹ-3ʹ)
*ACE*	Forward	5'-ATGGGGGCCGCGTCCGGCCA-3'
	Reverse	5'-TCAGGAGTGTCTGAGCTCCACC-3'
*Renin*	Forward	5'-ATGGGCGGGAGGAGGATGCC-3'
	Reverse	5'-TTAGCGGGCCAAGGCGAACCC-3'
*ACE2*	Forward	5'-ATGTCAAGCTCCTGCTGGCT-3'
	Reverse	5'-TTAGAATGAAGTTTGAGCATC-3'
*Ang II*	Forward	5'-ATGTGGCAGATTGTTTTCCT-3'
	Reverse	5'-TTAGAAATCTGCTGGCCGGA-3'
*Mas*	Forward	5'-TGACAGCCATCAGTGTGGAGA-3'
	Reverse	5'-GCATGAAAGTGCCCACAGGA-3'
*GAPDH*	Forward	5'-ATGGTGAAGGTCGGTGTGAACG-3'
	Reverse	5'-TTACTCCTTGGAGGCCATGTAGGC-3'

### Western blot analysis

Cardiac muscle tissue was homogenized in RIPA lysis buffer containing PMSF protease inhibitor. Protein concentration was determined with BCA protein assay kit. Then, 20 μg/lane protein samples were run in 12% sodium dodecyl sulfate polyacrylamide gel electrophoresis (SDS-PAGE) and transferred to PVDF membrane. The membrane was immunoblotted with antibodies after blocking with 5% skim milk in TBS containing 0.1% Tween-20 (TBST) at room temperature. The protein bands from diluted primary antibodies against ACE (1:500), ACE2 (1:400), renin (1:500), Ang II (1:500) and Ang-(1–7) (1:200) were normalized to the loading control, GAPDH. Blots were probed with HRP-conjugated goat anti-rabbit IgG and goat anti-mouse IgG for 1 h at room temperature followed by chemiluminescence detection.

### Statistical analysis

All data were expressed as mean values ± standard deviation (*n* ≥ 3). Data was analyzed by one way analysis of variance (ANOVA) with Tukey’s posthoc test. The PRISM 6.0 statistics software (Graph Pad Software, San Diego, CA) was used for all analysis. *p* < 0.05 was considered to be significant.

## Supplementary information

SUPPLEMENTAL MATERIAL

Rapeseed protein-derived peptides, LY, RALP and GHS, modulates key enzymes and intermediate products of renin-angiotensin system pathway in spontaneously hypertensive rat

## Data Availability

All data are included in this article

## Supplementary information

**Supplementary information** accompanies the paper on the *npj Science of Food* website (10.1038/s41538-018-0033-5).
