# Supplementary figures and images for: Rapeseed protein-derived peptides, LY, RALP, and GHS, modulates key enzymes and intermediate products of renin–angiotensin system pathway in spontaneously hypertensive rat
Source: NPJ Sci Food. 2019 Jan 17;3:1. doi: 10.1038/s41538-018-0033-5 (PMC6550218; doi:10.1038/s41538-018-0033-5)

ACE


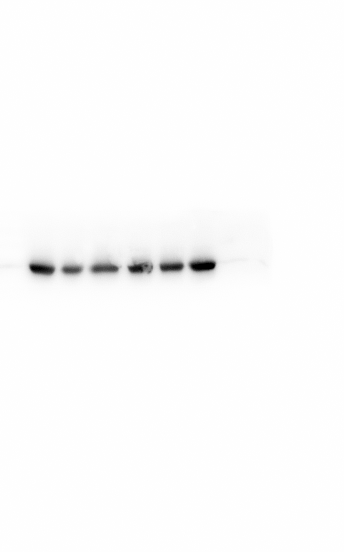


ACE2


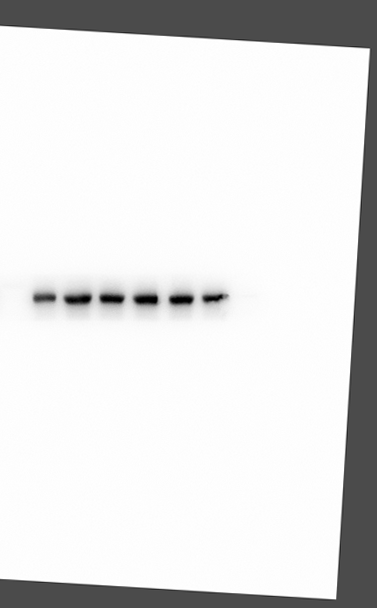


Ang II





ANG(1-7)





GAPDH


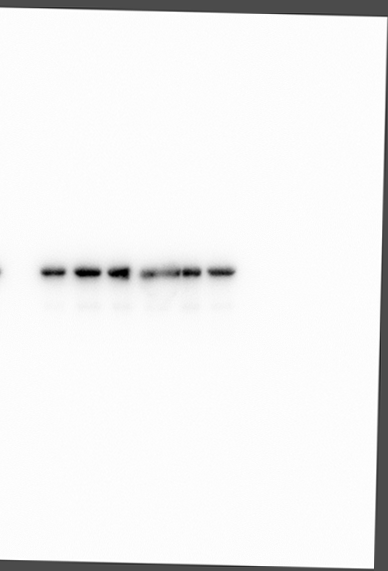


Renin

Supplement: Supplementary file 1 — SUPPLEMENTAL MATERIAL [file 41538_2018_33_MOESM1_ESM.docx]
