# Supplementary material for: Rapeseed protein-derived peptides, LY, RALP, and GHS, modulates key enzymes and intermediate products of renin–angiotensin system pathway in spontaneously hypertensive rat
Source: NPJ Sci Food. 2019 Jan 17;3:1. doi: 10.1038/s41538-018-0033-5 (PMC6550218; doi:10.1038/s41538-018-0033-5)

Supplementary data

Figure captions

Figure 1. Western blot analysis of the three peptides’ effects on renin, ACE, Ang II, ACE2 and Ang (1-7) in aorta tissue of SHRs compared with that of WKY rats. GAPDH was used as internal control.

Figure 2. Effects of three peptides LY, RALP and GHS on protein expression of ACE.

Figure 3. Effects of three peptides LY, RALP and GHS on protein expression of renin.

Figure 4. Effects of three peptides LY, RALP and GHS on protein expression of ACE2.

Figure 5. Effects of three peptides LY, RALP and GHS on protein expression of Ang II.

Figure 6. Effects of three peptides LY, RALP and GHS on protein expression of Ang-(1-7).

Figure 7. Effects of peptides on morphological changes in aorta in SHR and WKY

rats.

Figure 1


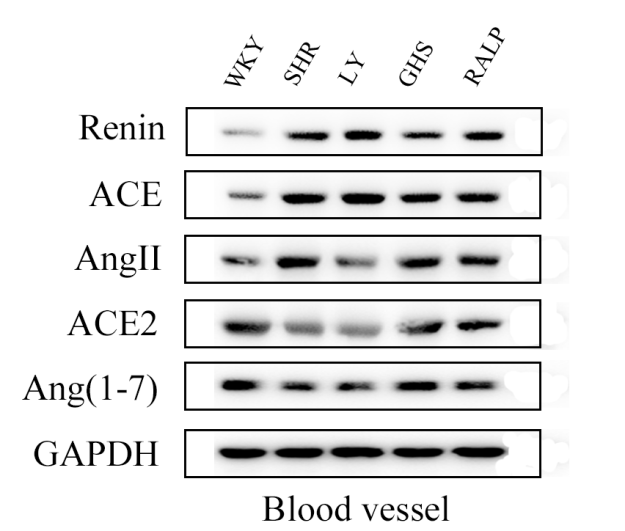


Figure 2


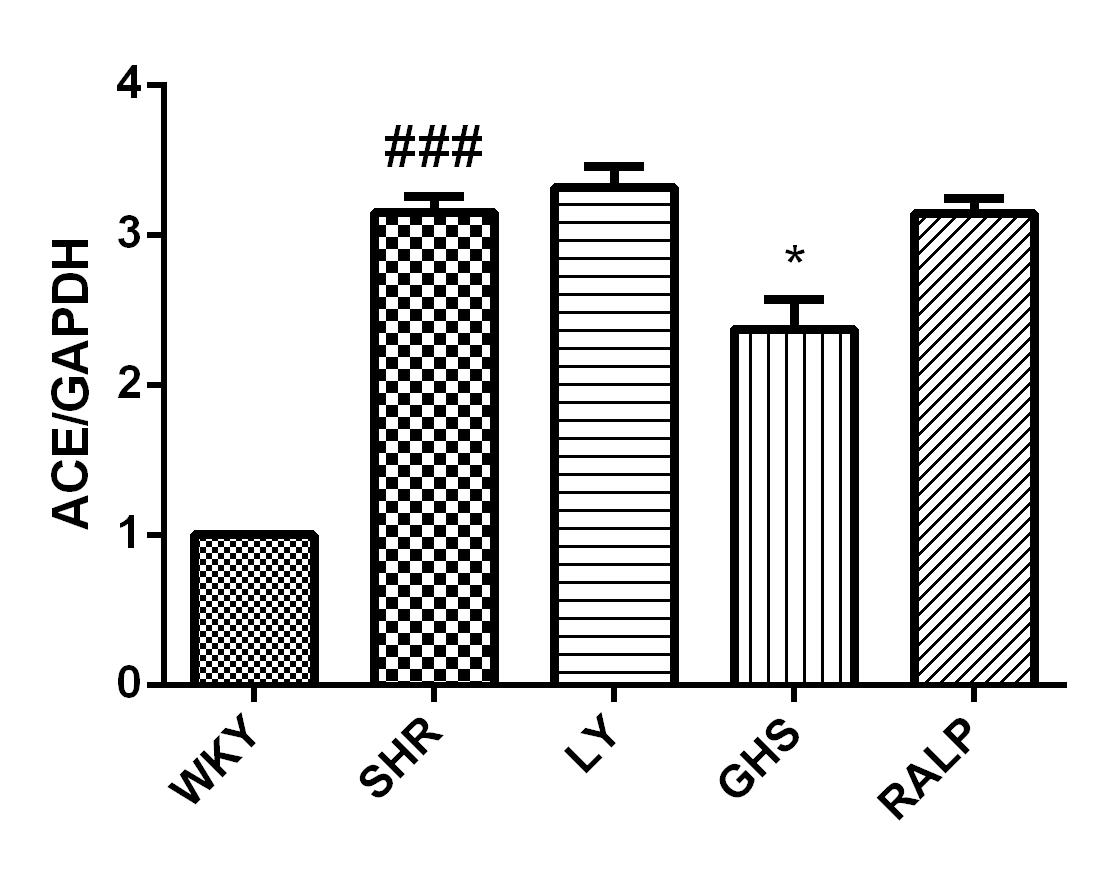


Figure 3


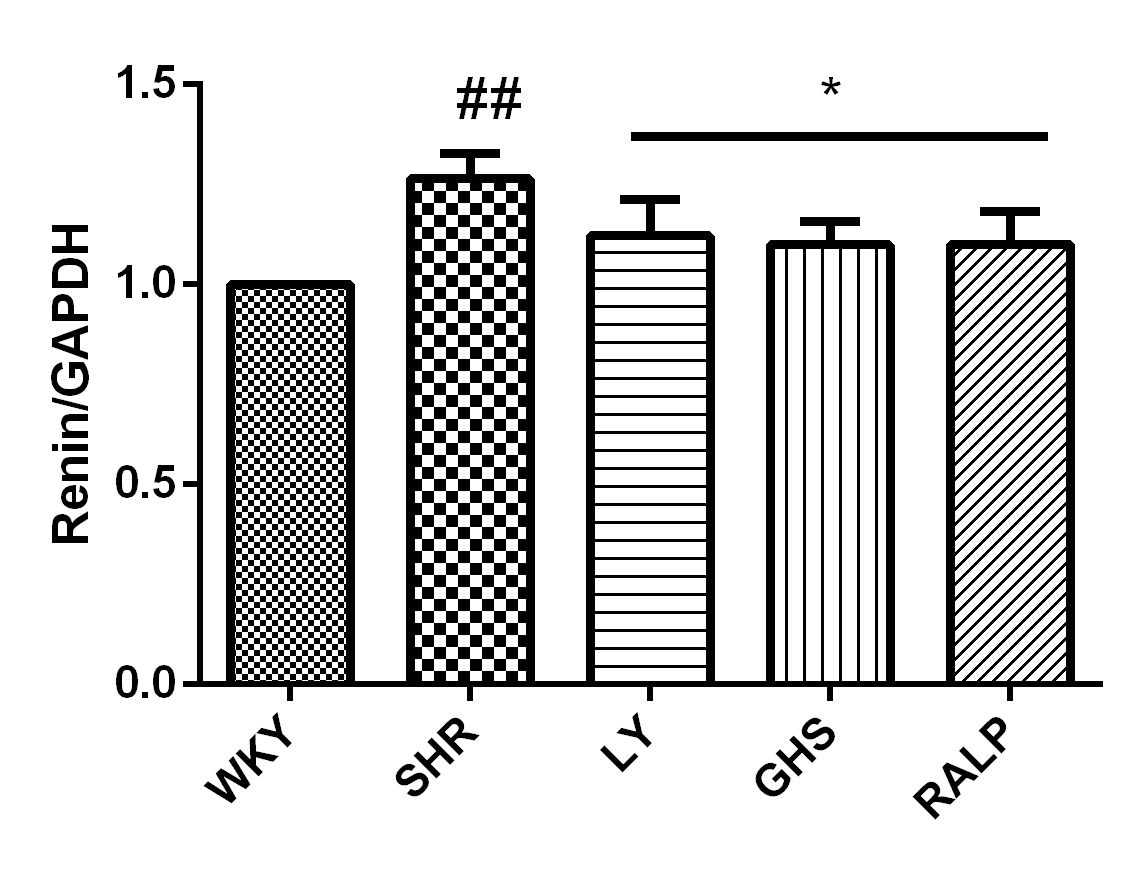


Figure 4


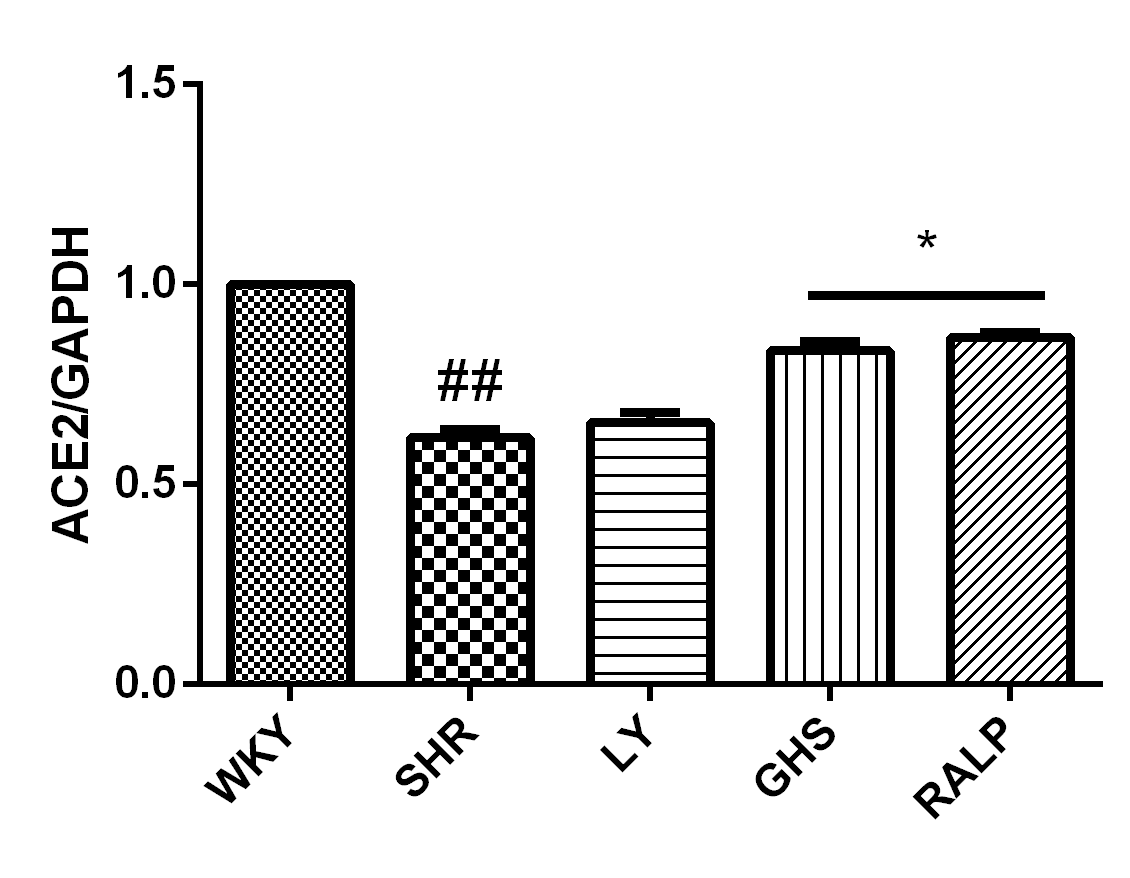


Figue 5


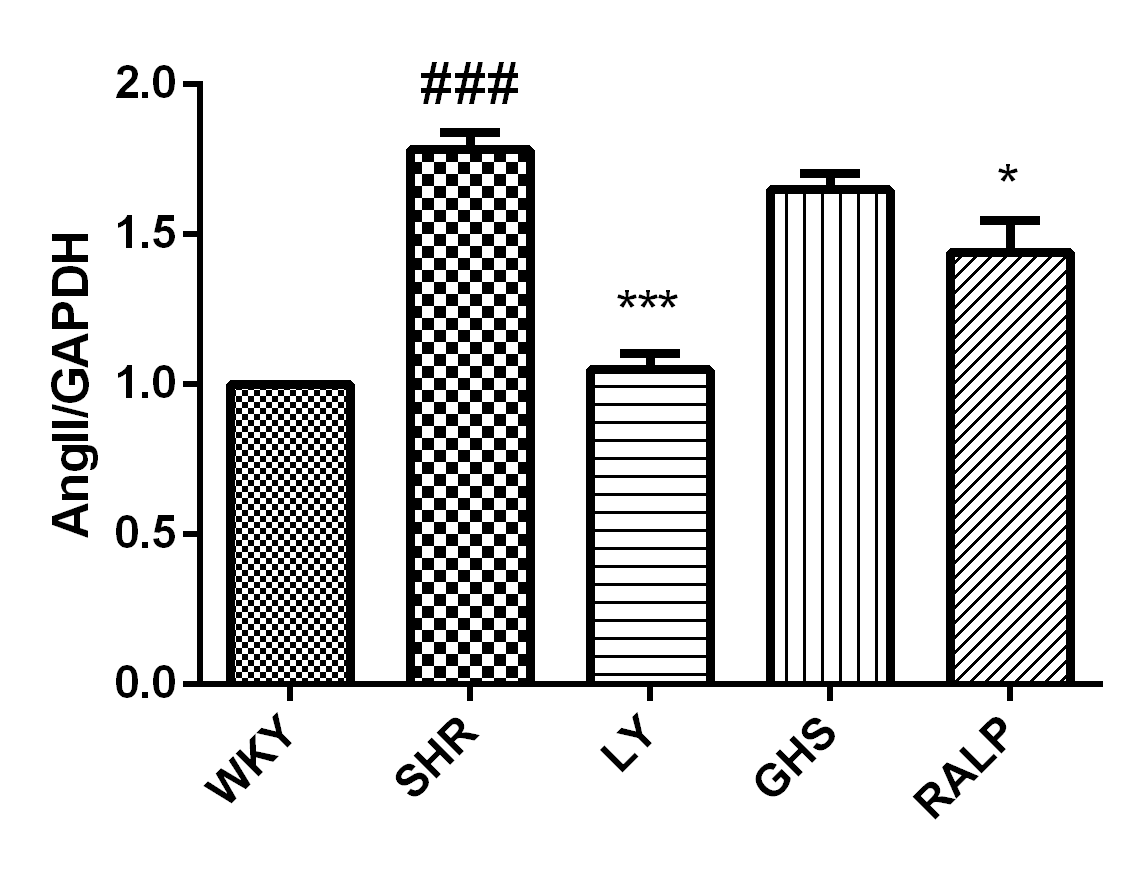


Figure 6


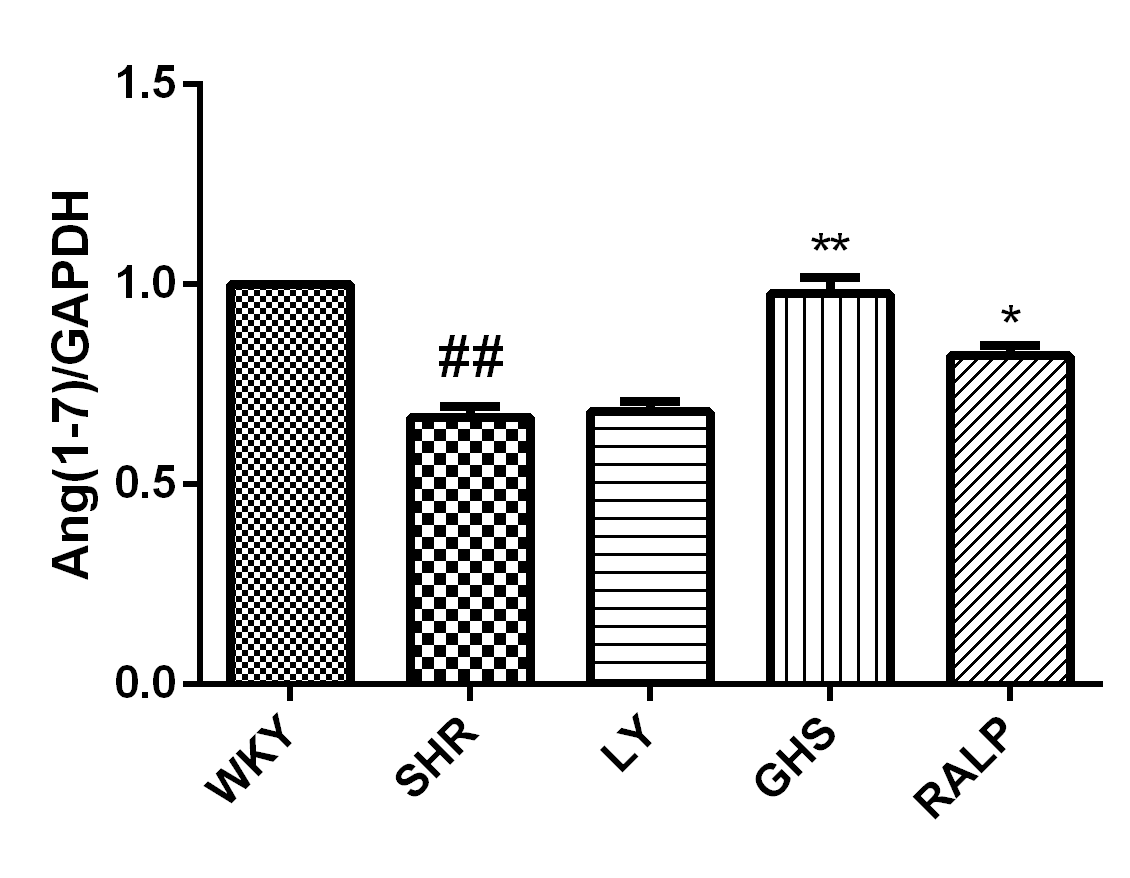


Figure 7


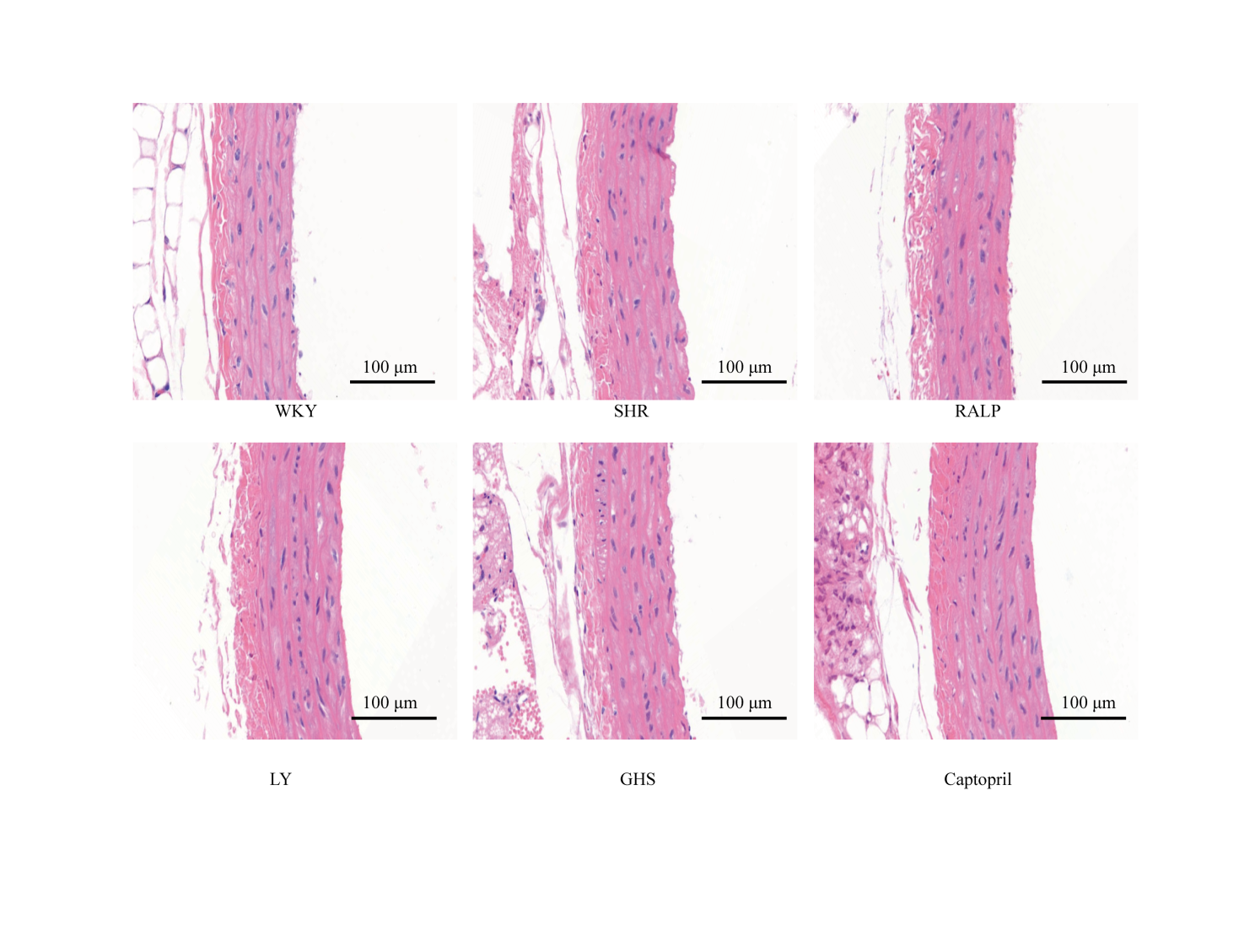

Supplement: Supplementary file 2 — Rapeseed protein-derived peptides, LY, RALP and GHS, modulates key enzymes and intermediate products of renin-angiotensin system pathway in spontaneously hypertensive rat [file 41538_2018_33_MOESM2_ESM.docx]
